# Breast Cancer and the Cardiovascular Disease: A Narrative Review

**DOI:** 10.7759/cureus.27917

**Published:** 2022-08-12

**Authors:** Swathi P Cherukuri, Rahul Chikatimalla, Thejaswi Dasaradhan, Jancy Koneti, Sai Gadde, Revanth Kalluru

**Affiliations:** 1 Research, Narayana Medical College, Nellore, IND; 2 Research, Kamineni Institute of Medical Sciences, Narketpally, IND

**Keywords:** endocrine therapy in breast cancer, breast cancer treatment, radiation therapy, anthracycline, heart and breast cancer, cardiovascular disease, breast cancer

## Abstract

Breast cancer is the most common malignancy affecting females worldwide and is also among the top causes of all cancer-related deaths. Cardiovascular disease (CVD) is known to have the highest rate of mortality in women. There are several risk factors for both CVD and breast cancer that overlap, such as diet, smoking, and obesity, and also the current breast cancer treatment has a significant detrimental effect on cardiovascular health in general. Patients with exposure to potentially cardiotoxic treatments, including anthracyclines, trastuzumab, and radiation therapy, are more likely to develop CVD than non-cancer controls. Early detection and treatment may reduce the risk of the development of cardiac morbidity and mortality and would increase the number of breast cancer survivors. This article provides a comprehensive overview of breast cancer, identifies shared risk factors among breast cancer and CVD, and the cardiotoxic effects of therapy. It also reviews possible prevention and treatment of CVD in breast cancer patients and reviews literature about chemoprevention of cardiac disease in the setting of breast cancer treatment.

## Introduction and background

Breast cancer is the most frequent cancer among women worldwide and also the leading cause of cancer-related death in women, with an estimation of 2.3 million cases and 685,000 deaths in 2020; in terms of incidence and mortality, it stands first in the majority of developed countries with an approximation of 24.5% of all cancer cases and 15.5% of all cancer deaths [1]. Estrogens and androgens in the bloodstream are linked to an increased risk of breast cancer [2]. Because of the heightened hormonal stimulation, female sex is one of the key variables linked to an increased risk of breast cancer, unlike men, who have low estrogen levels [3]. The risk of breast cancer increases with an increase in age, which is 1.5% risk at age 40, 3% at age 50, and more than 4% at age 70 [4]. Several genetic alterations are strongly associated with an elevated risk of breast cancer, of which BRCA1 (chromosome 17) and BRCA2 (chromosome 13) are the two important genes with great penetrance [5]. According to the cohort study conducted on 2.3 million women stated that the risk of breast cancer development is decreased in women with a gestation period lasting for 34 weeks and more when compared to women with a gestation period of 33 weeks and less [6]. Studies have shown that females who use hormonal replacement therapy (HRT) are at increased risk of breast cancer, and compared with never used, both estrogen-alone therapy and combined estrogen and progesterone therapy are linked to an elevated risk of cancer [7]. Breast cancer develops due to an inherent fault in deoxyribonucleic acid (DNA) and genes like P53, BRCA1, and BRCA2, along with exposure to estrogen, mutations in the genes encoding for the RAS/MEK/ERK pathway, and P13K/AKT pathway protect healthy cells from cell suicide. When the genes responsible for encoding these protective processes undergo mutation, the cells lose their ability to commit suicide when they are no longer needed, which then promotes the growth of cancer [8]. Also, the upregulation of the leptin pathway in breast promotes cancer growth by inhibiting apoptosis and by promoting angiogenesis [9]. The clinical presentation varies depending on the course of the disease; during the initial stages of the disease, the symptoms could be a hard painless lump and nipple discharge; as the disease progress, it could be characterized by lymph node involvement [10]. The breast cancer diagnosis can be made by taking patient history, breast examination and followed by diagnostic modalities like ultrasound imaging, mammography, nuclear medicine, single-photon emission computerized tomography (SPECT), positron emission tomography (PET), tumor markers like Ca 15-3, Ca 27.29, immunohistochemistry (IHC), fine-needle aspiration (FNA ), core biopsy and excisional biopsy are being used [8,10]. Surgery (lumpectomy, mastectomy, reconstructive surgery), radiational therapy (brachytherapy), hormonal therapy (anti-estrogen therapy, aromatase inhibitors), and chemotherapeutic drugs are the most common treatments for cancer in humans. Lumpectomy followed by radiational therapy is often used [8]. According to a study conducted among 63,566 women diagnosed with breast cancer, cardiovascular disease (CVD) was the primary cause of death in 15.9% of women. As the women aged and with different stages of breast cancer, the proportion of deaths due to cancer decreased, but the proportion of deaths due to CVD increased [11]. However, the public awareness of the coexistence of these two diseases is minimal, and CVD has become the major cause of death in US women [12]. This review article aims to emphasize the intersection of breast cancer and CVD by exploring the pathophysiological mechanisms of therapies of breast cancer that are leading to the development of CVD and explore the targeted therapies of breast cancer that could alleviate the incidence of CVD, and outline the preventive measures that could mitigate the development of CVD.

## Review

Breast cancer and CVD share several common risk factors. If left untreated, CVD poses a greater risk to health than cancer itself, so identifying and managing cardiovascular risk factors in this population is critical [13].

Risk of CVD in breast cancer therapy

Cancer treatment leads to early or delayed cardiotoxicity, ranging from left ventricular (LV) dysfunction, overt heart failure (HF), hypertension, arrhythmias, myocardial ischemia, valvular disease, and thromboembolic disease to pulmonary hypertension and pericarditis [14]. The time it takes for cardiotoxicity to develop varies significantly, with certain cancer treatments causing side effects that appear soon after exposure while others cause cardiac damage that manifests years later. Furthermore, some cancer therapies like anthracyclines might cause progressive cardiac remodeling as a late effect of previous myocyte destruction, leading to heart failure and may produce transitory cardiac dysfunction with no long-term repercussions in late cardiomyopathy, while others may cause acute cardiac dysfunction with long-term consequences [15].

Chemotherapeutic Drugs

Doxorubicin (DOX), an anthracycline, has traditionally been the drug of choice for treating breast cancer. It acts by intercalating and suppressing macromolecular production as well as topoisomerase II advancement in cardiac myocytes. Anthracyclines bind to topoisomerase II and interrupt the replication by interacting with DNA resulting in myocyte cell death [16]. Another most accepted mechanism is it increases the production of superoxide radicals, leading to oxidative stress and apoptosis of cells [17]. Topoisomerase II DNA topoisomerases cause transient single-stranded or double-stranded breaks to control topological changes during DNA replication, transcription, recombination, and chromatin remodeling [18]. Three distinct types of cardiotoxicity have been described with anthracycline therapy. Acute or subacute damage is an uncommon kind of cardiotoxicity that can occur within a week of receiving a single dose or a course of anthracycline medication in the form of pericarditis, myocarditis, or acute left ventricular failure [19]. Arrhythmias, such as ventricular, supraventricular, and junctional tachycardia, affect 0.5 to 3% of individuals with 0.7% total incidence [20]. Anthracyclines can also cause early-onset chronic progressive cardiotoxicity resulting in cardiomyopathy, which is more common and clinically significant and develops within a year after therapy is stopped and can lead to persistent dilated cardiomyopathy in adults and restrictive cardiomyopathy in children [21]. A longitudinal prospective cohort study of 277 breast cancer patients receiving doxorubicin suggested that, even after three years of anthracycline exposure, there is a moderate but persistent decline in left ventricular ejection fraction (LVEF) of 4% [22]. Early detection and treatment of LV dysfunction may result in LV functional recovery and a reduction in cardiac events. It should be noted that in no patient was there complete LVEF recovery seen following a time of more than six months after chemotherapy [23]. Dexrazoxane is a chelating drug that binds to intracellular iron, lowering the generation of free radicals and cardiomyocyte apoptosis, eliminating the DNA damage caused by doxorubicin in H9C2 cardiomyocytes [24]. Doxorubicin acts mainly by poisoning TOP2, producing ROS and, eliciting the DNA damage signal H2AX (Ser139 - phosphorylated H2AX, a crucial DNA damage signal induced by DNA double-strand breaks) in H9C2 cardiomyocytes. This doxorubicin-induced H2AX signal is fully suppressed in the presence of dexrazoxane (200mol/L) [24]. Dexrazoxane significantly lowers the incidence of anthracycline-induced congestive heart failure (CHF) and adverse cardiac events in women, regardless of whether the drug is given before the first dosage of anthracycline or the cumulative doxorubicin dose is ≥ 300 mg/m2 [25]. Clinical trials in women with advanced breast cancer have shown that patients are given dexrazoxane 30 minutes before doxorubicin (dexrazoxane to doxorubicin dosage ratio 20:1 or 10:1) have significantly lowered the overall incidence of cardiac events than placebo recipients (14 or 15% vs. 31%) [26]. A similar meta-analysis of seven trials found that dexrazoxane reduced cardiac events by 65% compared to placebo in 1000 patients [27]. Dexrazoxane is the only cardioprotective drug that has been shown to be effective in cancer patients receiving anthracycline chemotherapy, making it a viable choice for preventing cardiotoxicity in this patient group [25].

Alkylating Agents

Alkylating agents, such as cisplatin and cyclophosphamide, cause myocyte death and cytotoxicity by damaging DNA, with interstitial hemorrhage, edema, and necrosis being evidenced as a part of histopathology. Cyclophosphamide has been used most often in the treatment of breast cancer [14]. In 32 patients with hematologic malignant neoplasms, the cardiac effects of chemotherapy regimens containing high dosages of cyclophosphamide (180mg/kg over four days) were studied, of which nine individuals (28%) developed congestive heart failure within three weeks of receiving cyclophosphamide, with myocardial failure claiming the lives of six of these individuals (19%) [28]. A similar study in which a 53-year-old woman with cancer was administered cisplatin (37mg/m/wk) for three weeks [29]. The LVEF declined from 70% to 48% after discontinuing cisplatin and adding cardioprotective therapies like coenzyme Q10 (ubidecarenone, 10 mg TID) and trimetazidine (Vasorel, 20mg TID). Furthermore, the LVEF increased to 50% and 53% after 17 and 90 days, thus suggesting that cardioprotective agents minimize the drug-induced cardiovascular adverse effects and improve patient outcomes in the long-term use [29]. Along with coenzyme Q10 and trimetazidine, the acetyl-l-carnitine (ALCAR) was shown to be a protective agent for cisplatin-induced cardiotoxicity, as the superoxide dismutase-2 (SOD-2), which is a member of oxidant system expression was increased in cisplatin group but not in ALCAR (+) cisplatin group [30]. In addition to ALCAR, DL-α-lipoic acid and silymarin have also shown a protective potential against cisplatin-induced cardiotoxicity by decreasing reduced glutathione (GSH) contents and superoxide dismutase (SOD) activity [31].

Taxanes

Taxanes, such as paclitaxel, are effective for both early and advanced breast cancer. In the early stages, Taxanes can be given alone or in combination with anthracyclines. The ultrastructural appearance of the myocardium in a fatal instance of paclitaxel-induced cardiotoxicity is described, including sarcoplasmic reticulum enlargement, myofibril loss, lipofuscin accumulation, and laminated myeloid patterns [32]. On administration of paclitaxel, in phase two studies, asymptomatic sinus bradycardia was recorded in up to 29% of patients, and additional cardiac abnormalities such as atrioventricular conduction and bundles branch blockages, ventricular tachycardia, and potential ischemic symptoms were identified in about 3% of patients [33]. The administration of paclitaxel along with doxorubicin has become most common in the treatment of breast cancer, with paclitaxel aggravating the paclitaxel-induced cardiotoxicity by stimulating the anthracycline metabolites precipitating cardiac failure [34]. A dose-finding study conducted based on the pharmacokinetics of epirubicin and paclitaxel over three hours stated that the regimen with epirubicin 90mg/m2 and paclitaxel 200mg/m2 is the maximum tolerated dose (MTD) with low cardiotoxicity and high activity in metastatic breast cancer [35].

Antimetabolites

Antimetabolite drugs, like 5-fluorouracil (5-FU) and capecitabine, have been used as first-line for metastatic breast cancer and act by inhibiting the production of DNA and RNA [36]. They have been shown to cause cardiac effects in 1.2 to 7.6% of patients, and angina-like chest pain is recorded to be the most common symptom of fluorouracil-related cardiotoxicity [37]. Less common symptoms include cardiac arrhythmias, congestive cardiac failure, myocardial infarction, dilatative cardiomyopathy, cardiogenic shock, cardiac arrest, and sudden death syndrome [37]. A study was conducted on 1350 patients who were treated by 5-FU, of which 10 patients developed chest pain, gravitating anginal discomfort in three, EKG alterations and infarct-like pattern in two, heart failure in one with the coronary disorders resolving completely after the cessation of 5-FU [38]. Thrombosis or coronary arterial vasospasm has been suggested as a possible mechanism of chest pain [39]. The prevalence of cardiotoxicity with 5-FU depends on the dosage of the drug, a high dose of 5-FU could be the most common factor leading to acute and fatal visceral toxicity [40].

Endocrine Therapy

Hormonal therapy works by inhibiting the cellular processes through which estrogen supports the growth of normal and malignant tissue, and their cardiotoxicity is determined by the differences in their molecular targets [14].

Tamoxifen: Tamoxifen is a selective estrogen receptor (ER) modulator, which antagonizes the action of endogenous estrogens by competitively binding ER and by preventing E2-induced proliferation of breast cancer cells, thus inhibiting estrogen-dependent tumor growth [41]. A study suggested that tamoxifen significantly increased the high-density lipoprotein 2 (HDL2) by 47% and reduced the levels of low-density lipoprotein (LDL) and total serum cholesterol by 17% and 10%, respectively. In addition, it caused a 12% drop in apolipoprotein B, demonstrating a significant improvement in serum lipid profile, which could have a beneficial effect on the cardiovascular system [42]. However, clinical studies have not been able to show that tamoxifen has a protective advantage with respect to the cardiovascular system. And in contrast to its effect on lipids, tamoxifen causes a hypercoagulable state leading to thromboembolic complications, including both arterial and venous thrombosis in 5.4% of breast cancer patients [43]. Studies stated that tamoxifen has a low incidence of development of CVD (3.4%) but a high rate of thrombosis (2.8%) when compared to the aromatase inhibitors (AI) group having a high risk of development of CVD (4.2%) and low risk of thrombosis (1.6%) [44]. Also, tamoxifen reduces the recurrence rate of cancer, and the improvement in recurrence is more in the first five years of therapy, although the improvement in survival is larger in the first 10 years [45].

Aromatase inhibitors: Anastrozole, exemestane, and letrozole are third-generation aromatase inhibitors that have largely supplanted tamoxifen as the recommended treatment for hormone receptor-positive breast cancer in postmenopausal women [46]. After menopause, aromatase in muscle and fat may be the main source of circulating estrogen, and by reducing the activity of the aromatase enzyme, aromatase inhibitors and inactivators hinder the body’s ability to synthesize estrogen from androgens [46]. Estrogen exerts its protective effect on the cardiovascular system by regulating lipid levels, coagulation and fibrinolytic pathways, and synthesis of vasoactive molecules like nitric oxide and prostaglandins pathway [47]. Through nitric oxide and prostaglandins, estrogen promotes vasodilation and reduces atherosclerotic plaque formation [47]. As an AI-induced used reduction in circulating estrogens, as well as estrogen-mediated protective effects on the cardiovascular system, may result in an increased risk of CVD [48]. Although there were no significant differences in terms of cardiovascular events, the incidence of hypertension was considerably increased in the anastrozole group compared to placebo in a breast cancer prevention study with a follow-up of five years [49]. In the Arimidex, Tamoxifen, Alone or in Combination (ATAC) trial, after a median follow-up of 68 months, an increasing rate of hypercholesterolemia (9% vs. 3%) and hypertension (13% vs. 11%) was observed in the anastrozole group compared with tamoxifen group [50]. During endocrine therapy, statins, beta-blockers, angiotensin-converting enzyme inhibitors, sulfonylureas, and metformin could be used to treat hyperlipidemia, hypertension, and diabetes, which are recognized risk factors for CVD [48].

Targeted Therapies for Human Epidermal Growth Factor Receptor 2 (HER2) Positive Breast Cancer

Monoclonal antibodies like trastuzumab and pertuzumab are the two monoclonal antibodies currently approved by FDA to block human epidermal growth factor receptor 2 (HER2) signaling. The breast cancer population treated with trastuzumab has had the most extensive evaluation of LV dysfunction linked with targeted treatments [51]. Trastuzumab binds to the ErbB2 receptor tyrosine kinase 2/HER2, reduces signaling to the receptor through a variety of methods and it is widely believed that suppression of this receptor in the cardiac cells is leading to cardiac dysfunction [52]. The rate of CHF was discovered to be 29.4% among trastuzumab users compared to 18.9% among non-trastuzumab users in a cohort study of a total of 9,535 participants with a mean age of 71 years old, among which 2,203 (23.1%) received trastuzumab [52]. Among the patients receiving trastuzumab, the presence of cardiac risk factors like hypertension, coronary artery disease, and age more than 80 increases the risk of HF [53]. The APHINITY trial was conducted among node-positive or high-risk node-negative HER2 positive breast cancer women by comparing pertuzumab + trastuzumab with placebo + trastuzumab, and the incidence of primary cardiac events were 0.7% and 0.3 %, respectively, whereas the rate of decrease in LVEF is 0.6% and 0.2% [54]. Trastuzumab-induced cardiotoxicity (TIC) is reversible, unlike anthracycline-induced cardiotoxicity [55]. A variety of drug classes have been suggested to prevent TIC; the best-studied strategies include beta blockers and angiotensin-converting enzyme inhibitors (ACEi) [56]. Gujral et al. observed that using prophylactic beta blockers significantly reduced LVEF (p=0.02) and HF (p=0.01) among 1048 patients receiving anthracyclines with or without trastuzumab [57]. In a randomized controlled trial, Guglin et al. compared lisinopril, carvedilol, and placebo among 468 women with early HER2 positive breast cancer treated with trastuzumab for 12 months, the incidence of cardiotoxicity was 32% in the placebo group vs. 29% and 30% in carvedilol and lisinopril group, respectively, in the rest of the cohort (p=0.002). Both carvedilol (p=0.009) and lisinopril (p=0.015) effectively increased cardiotoxicity-free survival, indicating their cardioprotective effect on patients with risk of TIC [58]. Pertuzumab, emtansine, lapatinib, and neratinib are among the approved HER2 targeting breast cancer therapies to have a less detrimental effect on cardiac tissue than trastuzumab and to be more effective and safer in people with cardiac risk factors [56].

Different pivotal trials and rates of LVEF declines are discussed below for non-trastuzumab HER2-directed agents (Table 1). In a phase two randomized controlled trial (Neo-Sphere), trastuzumab plus docetaxel (group A), pertuzumab and trastuzumab plus docetaxel (group B), pertuzumab and trastuzumab (group C), or pertuzumab and docetaxel (group D) were all compared and LVEF decreased significantly in 1, 1, 3, and 1%, respectively [59]. A clinical trial of 3689 patients receiving lapatinib (monotherapy), either alone or in combination, shows a 1.6% rate of decline in LVEF [60]. In the other randomized trial of 324 patients with HER2 positive metastatic breast cancer, lapatinib plus capecitabine vs. capecitabine alone were compared, and LVEF decline was 2.4% and 0.7%, respectively [61]. In a randomized trial NEFERT-T of 479 patients where neratinib plus paclitaxel and trastuzumab plus paclitaxel were compared, and the decline in LVEF was 1.3% and 3.0% [62]. LVEF reduction was more common in the docetaxel plus trastuzumab plus placebo group than in the trastuzumab plus pertuzumab group (8.3% vs. 4.4%) in the CLEOPATRA trial [63].

**Table 1:** Different pivotal trials and rates of LVEF decline for non-trastuzumab HER2-directed agents PH - Perjeta (pertuzumab) + Herceptin (trastuzumab); TH - Taxotrene (docetaxel) + Herceptin (trastuzumab); TPH - Taxotere (docetaxel) + Perjeta (pertuzumab) + Herceptin (trastuzumab); HER2 - human epidermal growth factor receptor 2; LVEF - left ventricular ejection fraction

References	Trials	Number of patients	Population studied	Comparative arms	Rate of LVEF decline
Von Minkowitz et al. [54]	Pertuzumab (APHINITY)	4805	Patients with HER2-positive breast cancer (node-positive / high-risk node-negative)	PH	0.6%
				Placebo + trastuzumab	0.2%
Gianni et al. [59]	Pertuzumab (NEOSPHERE)	417	Patients with localized HER2-positive breast cancer	TH	0.9%
				TPH	2.8%
				PH	0.9%
				Docetaxel + pertuzumab	1.1%
Perez, et al. [60]	Lapatinib	3689	Patients with metastatic HER2-positive breast cancer	Lapatinib	1.6%
Geyer et al. [61]	Lapatinib	324	Patients with metastatic HER2-positive breast cancer	Lapatinib + capecitabine	0.7%
				Capecitabine	2.4%
Awada et al. [62]	Neratinib (NEFERT-T)	479	Patients with metastatic HER2-positive breast cancer	Neratinib + paclitaxel	1.3%
				Trastuzumab + paclitaxel	3.0%
Baselga et al. [63]	Pertuzumab (CLEOPATRA)	808	Patients with metastatic HER2-positive breast cancer	TPH	4.6%
				TH + placebo	7.4%

Emerging Therapies

Based on cross-talk between ER pathways, to overcome the endocrine resistance, cyclin-dependent kinase (CDK) 4/6 inhibitors are being explored [14]. CDK 4/6 is increased in cancer and contributes to tumor growth by inhibiting tumor suppression and apoptosis. Cell cycle disruption is caused by blocking the development of the CDK 4/6 cyclin D complex [64]. All three CDK 4/6 inhibitors - palbociclib, ribociclib, and abemaciclib, have undergone clinical studies and are used in combination with endocrine therapy to treat women with metastatic breast cancer [14]. Ribociclib is the only oral CDK 4/6 inhibitor associated with cardiovascular side effects: lengthening of QT interval was observed in 9% of patients at dosages of 600mg/d and 33% at doses >600 mg/d [65].

Radiation Therapy (RT)

Radiation damages the endothelial cells in the microvasculature, causing lymphocyte adherence and extravasation, resulting in thrombus development and capillary loss. Ischemia, myocardial cell death, and fibrosis come from a gradual decrease in capillary patency [66]. It produces inflammation and oxidative damage in large vessels like coronary and carotid arteries, which leads to lipid peroxidation and the production of foam cells, which starts the atherosclerotic process in the presence of excessive cholesterol [14]. Rapid atherosclerosis occurs as a result of radiation with thickened and fibrotic media/adventitia [67]. Chest RT holds a high risk of cardiac toxicity, which leads to high morbidity and mortality, limiting important cancer control and survival improvements [68]. Large case-control studies have strongly demonstrated a relationship between increased myocardial radiation dosage and major coronary complications. The calculated mean doses of radiation to the heart were, on average, 4.9 Gy (range 0.03 to 27.72), and when the mean radiation supplied to the heart increased by 1 Gy, the rate of major coronary events rose by 7.4 percent [69]. A study assessing the ischemic heart disease in breast cancer patients exposed to radiation found a 16.3% increase in the first four years while a 15.5% increase in the years following exposure after five to nine years, in comparison to subjects who did not receive radiation therapy (RT) at all [69].

Deep inspiration breath hold (DIBH) is an intervention to decrease CVD toxicity and is a procedure in which the patient is asked to take a deep breath when the dosage is delivered. This reduces the heart dose by shifting the heart towards the lungs and, with some intervening lung expansion leading to a decrease in cardiac tissue getting a considerable amount of radiation dose [70]. A study evaluating target coverage among 319 breast cancer patients stated that V20 Gy (the volume of the heart receiving a 20 Gy dose) changes from 7.8% to 2.3%, and V40 Gy (the volume of the heart receiving a 40 Gy dose) changes from 3.4% to 3.0% by decreasing the heart dose from 5.2 to 2.7 Gy and there was an increase in target coverage of the organ [71]. A study conducted among 33 patients stated that DIBH reduces cardiac mortality by 0.1 % and 4.8% with free breathing [72]. Alternative patient positioning has been studied extensively as a way to reduce cardiac exposure, and prone patient placement has been demonstrated to minimize cardiac dosage in patients with large pendulous breasts in clinical practice [73]. However, this does not apply to all patients, as the left anterior descending artery and the cardiac radiation doses were different in each position and were closely correlated with body mass index (BMI) [74]. When compared to the usual supine posture, a lot of studies have demonstrated that treating patients in a lateral decubitus position reduces cardiac doses. Bogart et al. stated that the mean heart doses for left-sided breast cancers ranged from 0.5 to 1.5 Gy and 0.25 to 0.52 Gy for right-sided breast cancers [75]. Proton therapy for breast cancer has demonstrated improved coverage of target volumes while keeping a low dosage to organs at risk, notably the heart itself, according to newer treatment modalities [76]. Cardiopulmonary radiation dose may be considerably reduced by proton beam RT. The physical characteristic of the radiation beam allows for the absence of an exit dose and precludes delivery of dose beyond the position of the target, resulting in the potential benefit of proton beam. When compared to conventional RT, proton beam RT for breast cancer exhibits low rates of toxicity and comparable rates of disease control [77].

Although proactive management of modifiable cardiac risk factors in the RT population has not been thoroughly explored, evidence from the general and high CV risk populations suggests that lowering these risk factors will also reduce cardiac morbidity in the future [78]. Risk classification and potential treatment in individuals receiving systemic breast cancer treatment are the first steps in developing primary prevention measures [79].

Role of beta blockers, renin-angiotensin-aldosterone blockade drugs, statins, and exercise in prevention

Prophylactic beta-blocker (BB) medication in breast cancer patients has been proven to be beneficial in small randomized, placebo-control trials. Prophylactic BB therapy (carvedilol or nebivolol) demonstrated less loss in LV function at six months than placebo before starting anthracycline-based chemotherapy in two trials, one of which included breast cancer patients mostly and the other of which included breast cancer patients only [80,81]. Another study indicated that continuous use of BB reduced the incidence of HF in breast cancer patients who progressed with anthracycline or trastuzumab breast cancer patients who were treated with anthracycline or trastuzumab [82]. In a randomized placebo control trial PRADA (prevention of cardiac dysfunction during adjuvant breast cancer therapy), candesartan, metoprolol succinate, and placebo were given to the breast cancer patients who had surgery and on chemotherapy as a part of the therapy. Candesartan prevented a small drop in LV function, while metoprolol succinate did not affect overall LVEF deterioration [83]. The other randomized placebo control trial, MANTICORE 101, included patients with HER2-positive early breast cancer and who were on trastuzumab as an adjuvant treatment. They also got medication with perindopril, bisoprolol, and placebo for the duration of adjuvant therapy (1:1:1). The placebo group experienced a minor drop in LVEF (5%), whereas the angiotensin-converting-enzyme (ACE) inhibitor group experienced a 3% drop and the BB group experienced a 1% drop in LVEF [84]. Enalapril and carvedilol were used to treat 201 patients with anthracycline-induced cardiomyopathy who had LVEF less than 45%. Forty-two percent of patients were considered responders and had moderate recovery in LVEF. LVEF recovery was predicted by a shorter time than HF therapy [23]. Studies demonstrating the prophylactic beta-blocker therapy are described below (Table 2).

**Table 2:** Studies demonstrating the prophylactic effect of beta blockers LVEF - left ventricular ejection fraction; LV - left ventricle; HF - heart failure; BB - beta-blockers; HER2 - human epidermal growth factor receptor 2

References	Design	Cases	Treatment aims	Diagnostic criteria	Conclusion
Kalay et al. [80]	Randomized controlled study	25 patients	Carvedilol vs. placebo	LVEF, systolic and diastolic	Prophylactic use of carvedilol in pts with anthracycline protects both systolic and diastolic functions of LV
Kaya et al. [81]	Prospective randomized controlled trial	45 patients with breast cancer	Nebivolol vs. placebo	Change in LVEF from baseline, N-terminal brain natriuretic peptide.	LVEF change; pre/post placebo: 66.6%/57.5%; nebivolol: 65.6%/63.8%. Nebivolol protects the myocardium against anthracycline-induced cardiotoxicity
Seitan et al. [82]	Follow-up study	920 patients with breast cancer	Beta-blockers	LVEF, HF incidence	Continuous use of BB lowers the incidence of HF in patients
Gulati et al. [83]	Randomized controlled study	130 women with breast cancer	Candesartan vs. metoprolol vs. candesartan+metoprolol	Change in LVEF on completion of adjuvant therapy	Mean LVEF % point reduction: placebo:2.6; candesartan:0.8; metoprolol:1.6. Concomitant treatment with candesartan protects against an early decline in LVEF
Pitkin et al. [84]	Randomized controlled study	33 petients with HER2-positive early breast cancer	Perindopril vs. bisoprolol vs. placebo	Change in LV volume and LVEF	No difference in the primary outcome
Cardinale et al. [23]	Clinical trial	201 patients with LVEF <45% due to anthracycline-induced cardiomyopathy	Enalapril vs. no treatment	Recovery in LVEF	Cardiotoxicity incidence control 25/58 (43%), enalapril 0/56 (0%)

ACE inhibitors have been the most well-researched drugs for preventing or treating LV dysfunction; spironolactone would be a protective drug given the rising importance of minerals corticoid antagonists in HF, particularly HF with maintained ejection fraction, and their capacity to reduce fibrous tissue [14]. A study has shown that when spironolactone was administered simultaneously along with anthracycline agents, a drop in ejection fraction was reduced in addition to diastolic stabilization [85].

Despite the lack of evidence that statins affect breast cancer incidence, there is some indication that they may improve breast cancer prognosis [14]. Simvastatin plays a therapeutic role in heart failure prevention by modulating antioxidant status and by inhibiting mitochondrial damage and cardiomyocyte apoptosis [86]. A Network meta-analysis of 22 relevant randomized controlled trials, including 1,916 patients with a mean age of 48.4 years, stated that the single drug of statin or in combination with spironolactone or enalapril has a significant cardioprotective effect than the placebo group [87]. Along with the cardioprotective effect, they can also reduce the recurrence rate, according to a prospective cohort study of early-stage breast cancer survivors stated that statins were associated with a lower risk of breast cancer recurrence (RR= 0.67;95 % CI,0.39 - 1,13), and with increased statin use, the risk of recurrence decreased [88].

An analysis of two prospective cohort studies, including 2973 women with nonmetastatic breast cancer, found a graded inverse relationship between exercise intensity and CV events in general. The benefit was seen in those who exercised for 10 metabolic equivalent hours per week, which is similar to the national exercise guidelines for adult cancer patients (nine metabolic equivalent hours per week ), and resulted in a 23% reduction in the risk of cardiovascular events, a 26% reduction in risk of CVD, and 29 % reduction in risk of HF [89]. Further clinical research is needed to determine whether exercise during cancer therapy is a realistic and beneficial technique for reducing cardiovascular morbidity and mortality in breast cancer survivors.

Limitations

We looked at several important pieces of research that may not have taken into account all of the data available for evaluation. Furthermore, our objectives did not include a detailed discussion regarding risk factors affecting CVD development and further surveillance.

## Conclusions

The prognosis of breast cancer is dependent on the presence of cardiovascular disease and the public is largely unaware of the cohabitation of these two diseases. Cardiovascular disease was the leading cause of mortality among women diagnosed with breast cancer, as cancer survivorship is largely influenced by the latent effects of CVD caused by cancer treatment. The similarity of predisposing risk factors in breast cancer and cardiovascular disease contributes significantly to the overlap between the two diseases, making it crucial to identify and manage cardiovascular risk factors in this population. The processes of cancer treatment related to cardiotoxicity have been better understood as a result of a holistic approach to the care of cancer patients receiving multimodality cancer therapy. Clinical approaches to lessen the negative effects of cancer treatments on cardiovascular health have been investigated and put into practice as a result. The results of the trials presented here show that the drugs used to treat heart failure with a lower ejection fraction could also be used to minimize cardiotoxicity in cancer therapy. These findings imply that neurohormonal blockade and beta-blockers have a minor impact on LVEF measured cardiac dysfunction decreases. Reduced troponin elevation and diastolic dysfunction have also been linked to these medicines. Breast cancer patients are a diverse group with unique cardiovascular and treatment-related risk factors, and the optimum primary prevention therapy plan at this time may include a combination of beta-blockers and neurohormonal medicines, either alone or in combination. In addition, a meta-analysis should be undertaken to establish which therapy is the most beneficial. More comprehensive data in this area may assist in further clarifying primary preventive treatment recommendations as greater risk subpopulations are explored. With the expanding convergence of the cardiovascular and oncologic fields, comprehensive care is becoming increasingly important in the management of cancer patients to optimize the advantages of cancer therapy while limiting the risk of cardiovascular health. The science of cardio-oncology to achieve the ultimate aim of lowering CVD morbidity and mortality in this expanding group. Advancement in characterizing the epidemiology, and pathophysiology of Cancer therapy-related cardiac dysfunction (CTRCD), improved risk stratification, and newer cardioprotective treatments are required for a better approach to the long-term management of the disease.
